# Evaluating the Risk of Hypophosphatemia with Ferric Carboxymaltose and the Recommended Approaches for Management: A Consensus Statement

**DOI:** 10.3390/jcm14144861

**Published:** 2025-06-27

**Authors:** Giuseppe Rosano, Justin Ezekowitz, Elizabeta Nemeth, Piotr Ponikowski, Martina Rauner, Melvin Seid, Donat R. Spahn, Jurgen Stein, Jay Wish, Robert J. Mentz

**Affiliations:** 1 Department of Human Sciences and Promotion of Quality of Life, San Raffaele Open University of Rome, 00166 Rome, Italy; 2 IRCCS San Raffaele, 00163 Rome, Italy; 3 Canadian VIGOUR Centre, University of Alberta, Edmonton, AB T6G 2E1, Canada; jae2@ualberta.ca; 4 Center for Iron Disorders, Department of Medicine, David Geffen School of Medicine, University of California, Los Angeles, CA 90095, USA; enemeth@mednet.ucla.edu; 5 Institute of Heart Diseases, Medical University and University Hospital, 50-367 Wroclaw, Poland; piotr.ponikowski@umw.edu.pl; 6 Division of Endocrinology, Diabetes and Bone Diseases, Department of Medicine III, and Center for Healthy Aging, University Medical Center, Technische Universität Dresden, 01062 Dresden, Germany; martina.rauner@ukdd.de; 7 Department of Obstetrics and Gynecology, University of Southern California Arcadia Hospital, Arcadia, CA 91007, USA; dr.melvinmd@gmail.com; 8 Faculty of Medicine, University of Zurich, 8091 Zurich, Switzerland; donat.spahn@swisspbm.ch; 9 Department of Gastroenterology and Clinical Nutrition, DGD Clinics Sachsenhausen, 60594 Frankfurt, Germany; j.stein@em.uni-frankfurt.de; 10 Division of Nephrology, Indiana University School of Medicine, Indianapolis, IN 46202, USA; jaywish@earthlink.net; 11 Duke University Medical Center and Duke Clinical Research Institute, Durham, NC 27710, USA; robert.mentz@duke.edu

**Keywords:** ferric carboxymaltose, hypophosphatemia, iron deficiency anemia, iron, phosphate

## Abstract

**Background/Objectives:** The development of hypophosphatemia has been associated with intravenous iron products, with the rate of hypophosphatemia found to be higher with ferric carboxymaltose. This consensus statement provides clinical guidance on the risk of hypophosphatemia development with ferric carboxymaltose and the approaches for management. To develop consensus recommendations regarding the clinical implications of hypophosphatemia after the administration of ferric carboxymaltose, the assessment of patient risk profile, and recommended approaches for risk reduction. **Methods:** Consensus statements were developed from an in-person meeting of specialists with expertise in iron pathophysiology and iron therapy and further supplemented with literature review. The multidisciplinary expert panel comprised global iron specialists spanning anesthesiology, cardiology, gastroenterology, obstetrics/gynecology, hematology, nephrology, and iron molecular biology. Structured discussions were held in an in-person meeting to gather expert opinion on the evidence base regarding intravenous iron and hypophosphatemia. Consolidated summary opinions underwent further iterations of panel review to form consensus recommendation statements. **Results:** The expert panel developed the following consensus statements: (1) Routine serum phosphate level measurement is not recommended for low-risk patients before or after treatment with ferric carboxymaltose, as most cases of hypophosphatemia that occur following the administration of ferric carboxymaltose are asymptomatic and transient; (2) patients receiving ferric carboxymaltose should be assessed for the degree of risk for developing symptomatic or severe hypophosphatemia prior to administration; (3) monitoring serum phosphate is recommended for patients at an increased risk for developing low serum phosphate or who require repeated courses of ferric carboxymaltose treatment at higher doses; (4) prophylactic oral phosphorus after ferric carboxymaltose is unlikely to effectively elevate phosphate and is not recommended for routine clinical practice; and (5) hypophosphatemic osteomalacia is rare and the risk of development after the administration of ferric carboxymaltose, in particular single infusion, is low. **Conclusions:** Hypophosphatemia following ferric carboxymaltose is predominantly asymptomatic and transient. Individuals at higher risk for developing hypophosphatemia with ferric carboxymaltose treatment include those who receive multiple infusions, higher cumulative doses, or long-term iron treatment or who have underlying clinical risk factors. These consensus statements provide structured guidance on the risk of hypophosphatemia with ferric carboxymaltose and the approaches to clinical management.

## 1. Introduction

Iron deficiency is a common condition worldwide and a leading contributor to the overall global burden of diseases [1,2]. A third of the world population is affected by anemia, largely caused by iron deficiency, with negative impact on patient morbidity, mortality, physical and mental function, and healthcare resource utilization [3]. Iron is a vital component of cellular processes, and absolute iron deficiency ensues when body iron stores are inadequate to meet physiological needs. Functional iron deficiency occurs when iron stores remain preserved yet there is insufficient plasma iron availability [4]. Both absolute and functional iron deficiencies are common occurrences in the adult population, even in the absence of anemia, particularly in individuals with heart failure, chronic kidney disease, or inflammatory disease [5,6].

The repletion of iron stores is a primary goal of treating iron deficiency, as well as normalizing hemoglobin levels in the setting of anemia [2]. Treatment approaches for iron deficiency and iron deficiency anemia, besides the treatment of the underlying medical condition, include both oral and intravenous iron formulations, with guidelines denoting that the choice of iron compound depends on the presence and degree of anemia, underlying cause of deficiency, clinical status, and/or patient preferences [7]. The use of intravenous iron is recommended for individuals who are unresponsive to oral iron, are intolerant to oral iron, or require rapid iron replenishment. Key patient populations recommended for intravenous iron treatment include those with chronic kidney disease, chronic heart failure, inflammatory bowel disease, intestinal malabsorption, after bariatric surgery, women with abnormal uterine bleeding, pregnant women with iron deficiency in the second or third trimester, or iron-refractory iron deficiency anemia [7,8,9,10,11,12,13,14].

Intravenous iron products feature a carbohydrate shell centered around an iron core [7,15]. Newer high-dose iron formulations comprise carbohydrate shells of greater stability, such as ferumoxytol, ferric carboxymaltose, and ferric derisomaltose, which allow for the administration of iron at greater doses, unlike iron sucrose or iron gluconate, which bind elemental iron less firmly [7,15]. Ferric carboxymaltose and ferric derisomaltose allow for high-dose iron delivery within a single administration or only a few administrations [7]. Low molecular weight iron dextran can be administered at high doses within a single administration but requires a test dose and an infusion time of 2 or more hours [7].

The established safety and tolerability profile of intravenous iron products has contributed to their increased use in clinical practice [15,16]. The development of hypophosphatemia has been associated with intravenous iron products, including iron dextran, ferumoxytol, ferric carboxymaltose, and ferric derisomaltose, although the rate of hypophosphatemia has been found to be higher with ferric carboxymaltose [17,18]. The incidence of hypophosphatemia following ferric carboxymaltose use has ranged from 2% to 85% in clinical trials and observational studies, depending on the patient population, dosing, and definitions utilized [19,20,21,22,23,24,25,26]. Separate pooled analyses of ferric carboxymaltose clinical trials determined that the rate of severe hypophosphatemia (serum phosphate level <1.0 mg/dL) was rare, occurring in 1% of patients [27,28].

The hypophosphatemic events after ferric carboxymaltose have generally been found to be asymptomatic and self-limited, with the majority of events resolving after approximately 8 weeks even without clinical intervention [7,25,27]. Post-marketing reports of the rate of hypophosphatemia after ferric carboxymaltose have been reflected in the updated label information from United States and European regulatory agencies [29,30]. A panel of iron specialists was convened to evaluate the clinical implications of hypophosphatemia after the administration of ferric carboxymaltose, identify patient risk profiles, and recommend approaches for risk reduction.

## 2. Materials and Methods

Consensus statements regarding hypophosphatemia and intravenous iron were developed following an in-person meeting of specialists with expertise in iron pathophysiology and iron therapy and further supplemented with a literature review. The multidisciplinary expert panel comprised global iron specialists spanning anesthesiology, cardiology, gastroenterology, obstetrics/gynecology, hematology, nephrology, and iron molecular biology. Structured discussions were held in an in-person meeting funded by CSL Vifor to gather expert opinion on the evidence base regarding intravenous iron and hypophosphatemia. A review of published literature was conducted via PubMed for all English-language articles related to ferric carboxymaltose and hypophosphatemia. The collected clinical insights were consolidated into core summary opinions, which underwent further iterations of panel review to form consensus recommendation statements (Table 1).

## 3. Results

### 3.1. Clinical Implications of Hypophosphatemia

The primary physiologic mechanisms for the development of hypophosphatemia are increased renal excretion, decreased intestinal absorption, and shifts of phosphate to intracellular compartments [31]. Common causes of acute hypophosphatemia more broadly include diabetic ketoacidosis, alcohol use disorder, severe burns, use of total parenteral nutrition, abdominal surgery, and refeeding syndrome from treatment of malnutrition [31,32,33,34]. Chronic hypophosphatemia, on the other hand, is typically due to renal or gastrointestinal losses of phosphate, such as from vitamin D deficiency, hyperparathyroidism, and Fanconi syndrome [31,32]. Certain genetic disorders also lead to chronic hypophosphatemia, such as X-linked hypophosphatemia, autosomal dominant hypophosphatemic rickets, autosomal recessive hypophosphatemic rickets, or hereditary hypophosphatemic rickets with hypercalciuria [31,35].

The normal values for serum phosphate concentrations in adults are 2.5–4.5 mg/dL (0.81–1.45 mmol/L) [36]. Serum phosphate levels below the reference range are categorized as mild hypophosphatemia at 2.0–<2.5 mg/dL (0.65–<0.81 mmol/L), moderate hypophosphatemia at 1.0–<2.0 mg/dL (0.32–<0.65 mmol/L), or severe hypophosphatemia at <1.0 mg/dL (<0.32 mmol/L) [32,36]. Notably, serum phosphate levels vary based on age, gender, and dietary intake, while also following a circadian rhythm with a morning nadir and afternoon peak [37,38].

Symptoms associated with hypophosphatemia are nonspecific and are more likely to occur with severe or chronic hypophosphatemia [39]. Mild hypophosphatemia typically is asymptomatic and often an incidental finding in up to 3% of general inpatients and 34% of intensive care patients [31,40]. Among hypophosphatemic patients who develop symptoms, the most common clinical presentations are muscle weakness and fatigue [31]. Neurological symptoms, such as paresthesias, dysarthria, confusion, or seizures, are rare but have been reported with severe hypophosphatemia [41,42]. Severe hypophosphatemia may also lead to musculoskeletal disorders, such as rhabdomyolysis and osteomalacia, or myocardial dysfunction [31,36,43]. Decreased myocardial contractility has been exhibited in patients with severe hypophosphatemia [33].

With intravenous iron products, the hypophosphatemia events are frequently mild or transient and are generally considered to have limited clinical impact on patients. The majority of hypophosphatemia cases after ferric carboxymaltose are asymptomatic [27,44,45,46,47,48]. However, cases of more severe hypophosphatemia with sequelae have been noted, particularly in those with an underlying risk of imbalanced phosphate metabolism, as discussed below [15]. In a retrospective study of patients treated with ferric carboxymaltose or iron sucrose, patients with moderate or severe hypophosphatemia were found to have no immediate clinical consequences, though some individuals experienced fatigue [49]. Similarly, a pooled analysis of the company-sponsored trials with ferric carboxymaltose found that 8.8% of subjects treated with ferric carboxymaltose had symptoms of hypophosphatemia and demonstrated no correlation between serum phosphate values and the occurrence of overall adverse events of hypophosphatemia. The study further noted the transience of hypophosphatemia after ferric carboxymaltose, with serum phosphate levels reaching a nadir at 2 weeks after administration and phosphate levels recovering by week 8 [27]. The transience of biochemical hypophosphatemia after ferric carboxymaltose administration has also been demonstrated in several studies of intravenous iron products, with comparable hypophosphatemia nadirs at 1 to 2 weeks after treatment and serum phosphate levels increasing in the weeks thereafter [44,45,46,50,51,52]. Given the evidence base for the mild and transient nature of hypophosphatemic events after ferric carboxymaltose administration, clinically relevant hypophosphatemia should be differentiated from asymptomatic hypophosphatemia, which may have limited clinical significance [15,36]. Patients receiving ferric carboxymaltose should be counseled regarding the symptoms of hypophosphatemia, especially if they need a number of treatments with iron over time at higher doses [29,30].

Consensus Statement 1: Routine serum phosphate level measurement is not recommended for low-risk patients before or after treatment with ferric carboxymaltose. Most cases of hypophosphatemia that occur following the administration of ferric carboxymaltose are asymptomatic and transient. For patients who develop symptoms, serum phosphate levels should be measured.

### 3.2. Hypophosphatemia Risk Evaluation

Decreases in serum phosphate have been demonstrated to arise more commonly after ferric carboxymaltose than other intravenous iron products [15,27]. Although most cases of hypophosphatemia have been found to be asymptomatic after ferric carboxymaltose administration, symptomatic hypophosphatemia events have been noted to occur in the post-marketing setting. Symptomatic hypophosphatemia, such as fatigue, myalgia, and bone pain in the setting of low serum phosphate, is more likely to occur in patients who have underlying medical risk factors, who receive multiple administrations at higher doses, or who have had long-term treatment [29,30]. Risk factors for the development of hypophosphatemia following ferric carboxymaltose include a history of gastrointestinal disorders associated with the malabsorption of fat-soluble vitamins or phosphate, concurrent or prior use of medications that affect proximal renal tubular function, hyperparathyroidism, vitamin D deficiency, malnutrition, and hereditary hemorrhagic telangiectasia [25,29,32]. A history of pre-existing hypophosphatemia also contributes to an increased risk of developing severe hypophosphatemia subsequent to treatment with ferric carboxymaltose [53,54,55].

Regulatory authorities recommend the monitoring of serum phosphate levels for patients who require multiple courses of treatment or those with existing risk factors for hypophosphatemia [29,30]. In the United States, a course of ferric carboxymaltose for adults weighing ≥50 kg is administered as two doses of 750 mg separated by at least 7 days for a total of 1500 mg of iron per course. An alternate dosage is 15 mg/kg body weight up to a maximum of 1000 mg as a single-dose treatment course. The European Medicines Agency-approved dosing regimen for ferric carboxymaltose is 1000 mg as a single dose, with a maximum of 1000 mg per week; additional doses should be a minimum of 7 days apart from the first dose [30]. The exposure to higher cumulative iron doses and the use of frequent multiple doses of ferric carboxymaltose with short dosing intervals are associated with greater reductions in serum phosphate levels [27,49,53]. Multiple courses of ferric carboxymaltose may lead to a stacking effect of hypophosphatemia risk, which may be addressed with the monitoring of serum phosphate levels in patients requiring several treatment courses [25].

The development of hypophosphatemia with ferric carboxymaltose is mediated by the upregulation of fibroblast growth factor 23 (FGF23), which is a phosphaturic hormone produced by osteocytes and a regulator of phosphate homeostasis [15,56,57]. Elevated FGF23 levels result in increased renal phosphate wasting, with ensuing reductions in serum phosphate [58]. The use of prophylactic phosphate supplementation after ferric carboxymaltose administration, however, was not found to prevent hypophosphatemia in a randomized controlled study comparing daily oral phosphate treatment to placebo [26]. Given that urinary phosphate excretion increases with oral phosphate loading [59], the administration of phosphate supplementation as a preventative measure is not recommended after ferric carboxymaltose infusions.

Several medications are associated with the development of hypophosphatemia, particularly bisphosphonates and diuretics [60,61]. Additional agents that may cause hypophosphatemia include carbonic anhydrase inhibitors, corticosteroids, acetaminophen overdose, antiandrogens, anticonvulsants, antineoplastics, mTOR inhibitors, calcineurin inhibitors, and alcohol [25,60,61,62]. The concomitant use of denosumab with ferric carboxymaltose has also been associated with an increased risk of developing hypophosphatemia [63,64]. The patient’s medication history should be reviewed before the administration of ferric carboxymaltose for the use of agents that may elevate the risk of hypophosphatemia (Table 2).

Patients with heart failure or chronic kidney disease have generally been found to have a low risk of developing moderate or severe hypophosphatemia after ferric carboxymaltose compared to other patient groups [27,28]. A retrospective study of iron-deficient patients with heart failure determined that 27% of patients developed hypophosphatemia following ferric carboxymaltose administration [65]. The clinically benign and transient nature of hypophosphatemia after ferric carboxymaltose in heart failure patients was also noted in a randomized controlled trial, which observed no long-term hypophosphatemia adverse events [66]; a sub-study of one trial found no events of symptomatic hypophosphatemia following ferric carboxymaltose [46,66]. Among individuals with chronic kidney disease, a low risk of hypophosphatemia among late-stage patients was mediated by the positive phosphate balance in chronic kidney disease due to dysregulation of phosphate reabsorption in renal proximal tubules and reduced phosphate excretion [67,68]. A single-center randomized controlled trial of intravenous iron products in patients with non-dialysis-dependent chronic kidney disease observed no events of moderate or severe hypophosphatemia subsequent to ferric carboxymaltose administration, with investigators noting that no clinically relevant hypophosphatemia events were observed [69]. Similarly, a pooled analysis of investigational trials of ferric carboxymaltose found post-treatment moderate to severe hypophosphatemia occurring in 12.8% of patients with non-dialysis-dependent chronic kidney disease and in no patients with hemodialysis-dependent chronic kidney disease [28].

For cases of mild or moderate hypophosphatemia after ferric carboxymaltose administration, treatment with phosphate is not necessary, and patients may be managed with watchful monitoring of symptoms [25,33]. We recommend that severe hypophosphatemia be managed according to the treatment protocols for phosphate supplementation per the clinic or hospital. In the event of persistent hypophosphatemia, treatment with ferric carboxymaltose should be re-evaluated [30].

**Figure 1:**
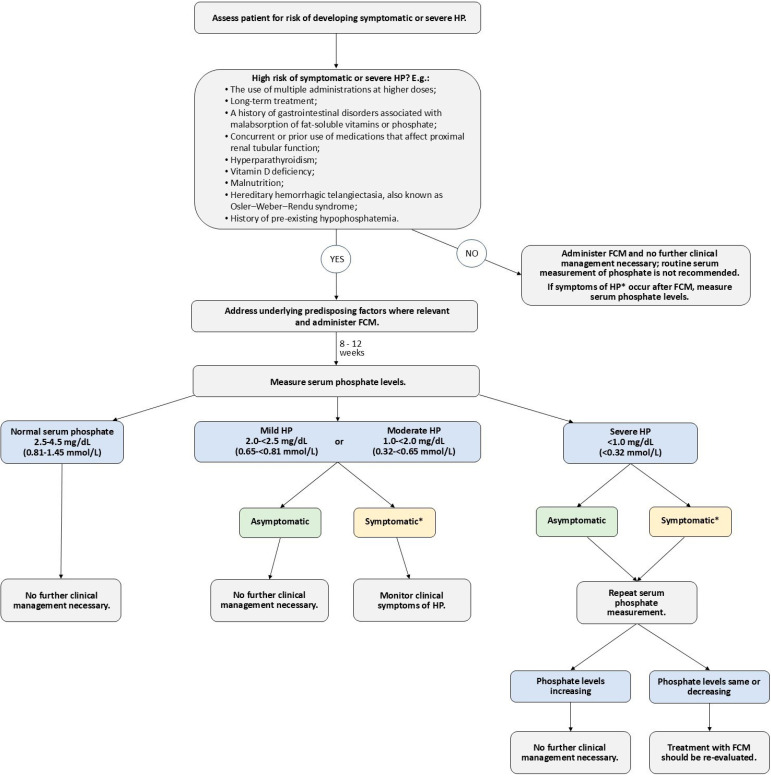
Approach to evaluating the risk of hypophosphatemia for patients treated with ferric carboxymaltose. * Symptoms include fatigue, myalgia, and bone pain. FCM, ferric carboxymaltose; HP, hypophosphatemia.

Consensus Statement 2: Patients receiving ferric carboxymaltose should be assessed for the degree of risk for developing symptomatic or severe hypophosphatemia prior to administration. For those at risk of developing clinically relevant hypophosphatemia, underlying predisposing factors should be addressed prior to initiating ferric carboxymaltose treatment. Factors that elevate the risk of symptomatic or severe hypophosphatemia following ferric carboxymaltose include the use of multiple administrations at higher doses, long-term treatment, a history of gastrointestinal disorders associated with malabsorption of fat-soluble vitamins or phosphate, concurrent or prior use of medications that affect proximal renal tubular function, hyperparathyroidism, vitamin D deficiency, malnutrition, hereditary hemorrhagic telangiectasia (also known as Osler–Weber–Rendu syndrome), and a history of pre-existing hypophosphatemia (Figure 1).

Consensus Statement 3: Monitoring serum phosphate is recommended for patients at increased risk for developing low serum phosphate or who require repeated courses of ferric carboxymaltose treatment at higher doses.

Consensus Statement 4: Prophylactic oral phosphorus after ferric carboxymaltose is unlikely to effectively elevate phosphate and is not recommended for routine clinical practice.

### 3.3. Clinical Sequelae of Hypophosphatemia

Although hypophosphatemia events following ferric carboxymaltose administration are predominantly transient, low serum phosphate levels may persist in some patients after treatment. Chronic hypophosphatemia may lead to further clinical sequelae, such as osteomalacia [31]. In the setting of acute hypophosphatemia, however, the likelihood of developing osteomalacia is low given the duration of time involved to progress through the stages of disease pathophysiology, advancing from hypophosphatemia to bone mineralization defects before the development of osteomalacia [31,70].

The diagnosis of osteomalacia is established by specific clinical criteria, which include hypophosphatemia or hypocalcemia, elevated bone alkaline phosphatase, muscle weakness or bone pain, bone mineral density <80% in young adults, and imaging findings of Looser zones or multiple uptake zones by bone scintigraphy [62,71]. An alternate set of criteria for the diagnosis of osteomalacia for patients without liver or kidney disease is the presence of elevated parathyroid hormone, elevated total alkaline phosphatase, low urinary calcium, and either low calcidiol levels (<30 nmol/L) or low calcium intake (<300 mg per day) [62,72]. The clinical presentation of osteomalacia may include bone pain, muscle weakness, or fragility fractures [62,70]. Although epidemiologic data on osteomalacia are limited, an evaluation of bone histology in adult Europeans determined the prevalence of osteomalacia to be 25.6% in the study population [73].

Rare cases of hypophosphatemic osteomalacia after the administration of ferric carboxymaltose have been reported in the post-marketing setting [29,30]. The majority of case reports have reflected patients who received multiple doses of ferric carboxymaltose over a prolonged time period or had underlying medical conditions associated with lower phosphate levels at baseline independent of exposure to ferric carboxymaltose [25,74,75,76]. Correspondingly, the consensus panel experts have not observed an elevated rate of bone disease after treatment with ferric carboxymaltose in their clinical practices. Given the predominantly transient nature of hypophosphatemia after ferric carboxymaltose administration, the development of osteomalacia or subsequent bone fractures is unlikely after a single course of ferric carboxymaltose or in the setting of acute hypophosphatemia.

Consensus Statement 5: Hypophosphatemic osteomalacia is a rare occurrence, and the risk of development after the administration of ferric carboxymaltose, in particular single injections, is low.

## 4. Discussion

The development of hypophosphatemia has been reported as a side effect that takes place more frequently with ferric carboxymaltose than with other intravenous iron products. However, biochemical hypophosphatemia events following ferric carboxymaltose are predominantly asymptomatic and transient, typically improving within eight weeks, with severe hypophosphatemia being a rare occurrence. Given this mild and self-resolving course, routine serum phosphate level measurements are not recommended for all patients treated with ferric carboxymaltose, in contrast to alternate proposed algorithms advising phosphate surveillance [77].

Individuals who are at a higher risk for developing hypophosphatemia with ferric carboxymaltose treatment should be monitored and include those who receive multiple infusions, higher cumulative doses, or long-term iron treatment or who have underlying clinical risk factors. Such factors include a history of gastrointestinal disorders associated with malabsorption of fat-soluble vitamins or phosphate, concurrent or prior use of medications that affect proximal renal tubular function, hyperparathyroidism, vitamin D deficiency, malnutrition, hereditary hemorrhagic telangiectasia, or pre-existing baseline hypophosphatemia. Concomitant medications may further contribute to the risk of developing hypophosphatemia, particularly bisphosphonates, diuretics, and denosumab.

Although acute hypophosphatemic events after ferric carboxymaltose are typically mild with minimal clinical impact, prolonged hypophosphatemia may lead to osteomalacia. Cases of hypophosphatemic osteomalacia subsequent to ferric carboxymaltose have been rare, with the majority of reported patients having contributory underlying medical conditions or a history of prolonged exposure to ferric carboxymaltose. The benefit–risk profile of ferric carboxymaltose has been further reflected in the over 33 million patient-years of exposure to ferric carboxymaltose in the post-marketing setting [78] and the demonstrated benefits to patient quality of life [79,80]. For patients treated with ferric carboxymaltose, routine serum phosphate testing would not be recommended for the general patient population; however, patients identified to be at a greater risk for hypophosphatemia would benefit from the monitoring of serum phosphate levels after treatment with ferric carboxymaltose.

## Figures and Tables

**Table 1:** Expert panel consensus regarding ferric carboxymaltose and hypophosphatemia.

Consensus Statements
Routine serum phosphate level measurement is not recommended for low-risk patients before or after treatment with ferric carboxymaltose. Most cases of hypophosphatemia that occur following the administration of ferric carboxymaltose are asymptomatic and transient. For patients who develop symptoms, serum phosphate levels should be measured.
2. Patients receiving ferric carboxymaltose should be assessed for the degree of risk for developing symptomatic or severe hypophosphatemia prior to administration. For those at risk of developing clinically relevant hypophosphatemia, underlying predisposing factors should be addressed prior to initiating ferric carboxymaltose treatment. Factors that elevate the risk of symptomatic or severe hypophosphatemia following ferric carboxymaltose include - The use of multiple administrations at higher doses; - Long-term treatment; - A history of gastrointestinal disorders associated with malabsorption of fat-soluble vitamins or phosphate; - Concurrent or prior use of medications that affect proximal renal tubular function; - Hyperparathyroidism; - Vitamin D deficiency; - Malnutrition; - Hereditary hemorrhagic telangiectasia, also known as Osler–Weber–Rendu syndrome; - A history of pre-existing hypophosphatemia.
3. Monitoring of serum phosphate is recommended for patients at increased risk for developing low serum phosphate or who require repeated courses of ferric carboxymaltose treatment at higher doses.
4. Prophylactic oral phosphorus after ferric carboxymaltose is unlikely to effectively elevate phosphate and is not recommended for routine clinical practice.
5. Hypophosphatemic osteomalacia is a rare occurrence, and the risk of development after the administration of ferric carboxymaltose, in particular single injections, is low.

**Table 2:** Drugs associated with elevated risk of developing hypophosphatemia.

Bisphosphonates Denosumab Diuretics Carbonic anhydrase inhibitors Corticosteroids Acetaminophen overdose Anti-androgens Anticonvulsants Antineoplastics mTOR inhibitors Calcineurin inhibitors Alcohol use
